# Can a Commercial Video Game Prevent Depression? Null Results and Whole Sample Action Mechanisms in a Randomized Controlled Trial

**DOI:** 10.3389/fpsyg.2020.575962

**Published:** 2021-01-12

**Authors:** Marlou Poppelaars, Anna Lichtwarck-Aschoff, Roy Otten, Isabela Granic

**Affiliations:** ^1^Radboud University, Behavioural Science Institute, Nijmegen, Netherlands; ^2^Research and Development, Pluryn, Nijmegen, Netherlands; ^3^National Institute on Drug Abuse, ASU REACH Institute, Arizona State University, Tempe, AZ, United States

**Keywords:** prevention, randomized controlled trial, depression, adolescents, young adults, commercial video games, journey, flower

## Abstract

Depressive symptoms and disorders are major public health concerns, affecting many adolescents and young adults. Despite extensive research, depression prevention programs for youth show limited effectiveness. Moreover, the maximal potential of youth psychotherapy — on which depression prevention programs are based — may have been reached. Commercial video games may offer an engaging alternative vehicle for youth to practice emotional and social skills vital to mental health. The current study investigated the potential for the commercial video game Journey to prevent the exacerbation of depressive symptoms. A pre-registered randomized controlled trial tested the effectiveness of Journey as an indicated depression prevention approach compared to a control game condition and a passive control condition (Dutch Trial Register: NL4873, https://www.trialregister.nl/trial/4873). Additionally, potential action mechanisms for depression prevention using video games were examined. Participants aged 15 to 20 years old with elevated depressive symptoms (*n* = 244, *M*_*age*_ = 17.11, *SD*_*age*_ = 1.76, 66.4% female) were given 4 weeks to play Journey (*M*_*duration*_ = 3 h 20 min) or the control game, Flower (*M*_*duration*_ = 2 h 36 min). Results showed no beneficial effects of playing the commercial video game, Journey, on youth’s change in depressive symptoms above and beyond the active and passive control conditions up to 12-months after the intervention. Additionally, no action mechanisms were found specifically for Journey. Nevertheless, over the whole study, participants decreased in depressive symptoms, became less sensitive to rejection, and experienced more hope and optimism. Moreover, participants who during the study decreased in rejection sensitivity or rumination or who increased in hope and optimism or in distraction and problem solving showed the strongest decrease in depressive symptoms. Although results do not support the use of the studied commercial game as an effective indicated depression prevention strategy, our results do suggest that rejection sensitivity, hope, optimism, rumination, distraction, and problem solving are promising targets for future depression prevention efforts. We conclude with important lessons for future research on games to promote mental health. Particularly, encouraging careful consideration of research designs to explore for whom and how potential action mechanisms and associated game mechanics may be effective.

## Introduction

Both depressive symptoms and depressive disorders are major public health concerns, negatively impacting individuals’ achievements, social interactions, and future mental health (Wesselhoeft et al., 2013; Hetrick et al., 2016; Carrellas et al., 2017). During adolescence and young adulthood individuals are most at risk for the emergence of depression (Merikangas et al., 2010; Avenevoli et al., 2015), with over 10% of youth experiencing depression in the past year (Weinberger et al., 2018). Unsurprisingly, research on depression prevention programs for youth is extensive (e.g., Horowitz and Garber, 2006; Merry et al., 2011; Hetrick et al., 2016). However, effect sizes are limited and even indicated prevention programs may not be superior to attention control groups (Hetrick et al., 2016). Moreover, a recent meta-analysis shows that the maximal benefit of youth psychotherapy — on which depression prevention programs are based — has been reached (Jones et al., 2019), suggesting the need for genuine innovations in content and delivery of prevention approaches (Kazdin, 2019). In an attempt to explore alternatives to traditional depression prevention in youth, this study tested the effectiveness of a commercial video game hypothesized to affect a number of empirically supported action mechanisms.

In contrast, most traditional depression prevention programs are based on cognitive behavioral therapy (Hetrick et al., 2016). Cognitive behavioral therapy assumes that depression results from cognitive distortions that lead a person to see themselves, their environment, and their future negatively (Beck, 1976). These biases are associated with learned helplessness in which individuals withdraw or react passively due to their sense of not being in control of negative events (Seligman et al., 1979). Many traditional depression prevention programs use psychoeducation, written exercises, role-playing exercises, and homework assignments to address these cognitive distortions and their associated behaviors (e.g., the Penn Resiliency Program; Gillham et al., 2006; Hetrick et al., 2016).

These prevention programs are often didactic and highly cognitive group versions of depression treatment (Hetrick et al., 2016), which limits their appeal for youth. Digitalization of these programs into websites and serious games is sometimes seen as a solution to enhance effectiveness through engagement and personalization (Scholten and Granic, 2019). This form of digitalization, however, has not enhanced effectiveness and in fact may be less effective without human guidance (for review, see Scholten and Granic, 2019). Rather than appealing to youth and utilizing options for interaction and personalization, digitized programs are associated with considerable program attrition, leading researchers to call for critical reflection on engagement when developing these interventions (Välimäki et al., 2017; Garrido et al., 2019; Scholten and Granic, 2019).

Commercial video games in contrast are highly effective in engaging youth for multiple hours a week and maintaining this engagement (Pew Research Center, 2018; Limelight Networks, 2019). And while commercial games are not designed to address mental health, limited research has shown beneficial effects of commercial video games on depressive symptoms (Ferguson and Rueda, 2010; Russoniello et al., 2013). Specifically, a randomized controlled trial showed a decrease in depressive symptoms in adults with elevated depressive symptoms when they played a casual video game (i.e., a quick, fun, and accessible game) three times a week for 30 min during 1 month compared to an active control group (Russoniello et al., 2013). This effect was attributed to the introduction of a pleasant activity. Indeed, the mere experience of motivation and engagement which video games evoke may support mental health benefits through flow (immersion in an activity that engages all your attention), intrinsic motivation, experienced autonomy (i.e., the freedom to choose), and competence (i.e., the experience of capability to overcome challenges) which have all been linked to mental health benefits (see Ryan et al., 2006; Nakamura and Csikszentmihalyi, 2009; Zuroff et al., 2012).

Furthermore, commercial video games can possess a number of additional characteristics that may enhance their potential to improve mental health (Granic et al., 2014). Intense emotions which can be evoked by narrative techniques, may allow youth to practice coping strategies while facing negative emotions in a safe environment (Granic et al., 2014). Similarly, the social nature of video games allows for a relevant practice of social interactions.

**FIGURE 1:**
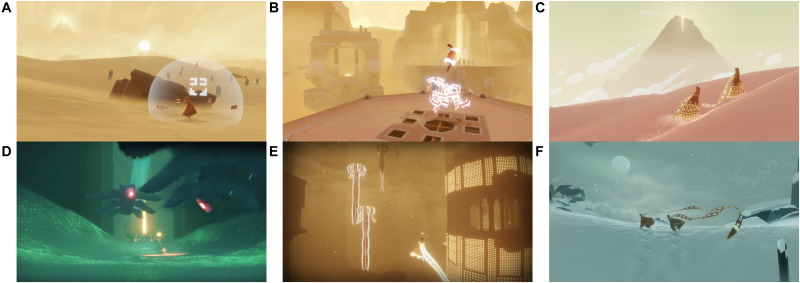
Gameplay in Journey. Players travel through the desert **(A–C)** and underground areas **(D,E)** to the top of a snowy mountain **(F)**. Players communicate with a single tone **(A)** and can fly if energized by cloth **(B)** or other players **(C)**. These images are reproduced from Journey by Thatgamecompany (2012b) with the permission of the copyright holder Sony Interactive Entertainment.

Journey (Thatgamecompany, 2012b) is a commercial game that has been praised for eliciting intense emotions, its visual beauty, and unique social gameplay (Metacritic, 2012; Thatgamecompany, 2012a). Essentially the game chronicles the journey of the player’s avatar from a desert wasteland to a distant mountain in eight levels (see Figure 1)^1^. Interestingly, Journey was designed to evoke feelings of being relatively powerless (Lowthorpe and Taylor, 2017), allowing the player to experience fear and a struggle for freedom. Additionally, anecdotal evidence in the form of player accounts linked Journey to overcoming grief, loneliness, and depressive episodes (e.g., Asgeek2012, 2012; Journey Stories, 2012). Rather than designing game mechanics of a serious game using evidence based action mechanisms (e.g., exposure; see for an example Scholten and Granic, 2019), the anecdotal evidence inspired us to reverse this process and identify game mechanics which could affect empirically supported action mechanisms. Specifically, we hypothesized that Journey may prevent the exacerbation of depressive symptoms through the game’s social interaction, narrative, and quick succession of negatively and positively valenced scenes affecting four mechanisms: (a) rejection sensitivity, (b) narrative identity, (c) hope, and (d) coping strategies. The research supporting each action mechanism and their potential to improve depressive symptoms is reviewed below, with a rationale of how Journey’s game mechanics may impact depressive symptoms through each mechanism.

First, research has suggested that rejection sensitivity – the anticipation and perception of rejection followed by a strong emotional reaction to perceived rejection – underlies deficits in social skills in depression and is a maintaining factor for depressive symptoms (Marston et al., 2010). Interestingly, video gameplay and particularly cooperative gameplay was found to influence elements of social competence in a number of studies (Gentile et al., 2009; Greitemeyer et al., 2010). In Journey specifically, players are encouraged to bond together through a mechanic that makes them more powerful when they are close to another player. The cooperative play leaves no room for explicit negativity between players, yet allows for very limited communication open to interpretation (see Figure 1A). Thus, when players are disconnected from another, this unexpected loss can feel compelling (e.g., Lowthorpe and Taylor, 2017), particularly for players who are rejection sensitive. A repeated confrontation with ambiguous rejection and reconnection with another player may allow players to reinterpret the loss experience as less catastrophic or personal, potentially re-training their interpretation of rejection.

Next, narrative identity and feelings of hope and optimism may be additional action mechanisms in the game which are promoted by the same game mechanics. Narrative identity — a person’s life story that provides them with meaning and coherence — has been theorized to be essential for mental health (McAdams and McLean, 2013). Research on narrative development has shown that those who ascribe more redemptive meaning to suffering in their narrative identity (i.e., negative events are redeemed by causing or being followed by a positive event) have greater mental health and those finding more examples of personal agency over the course of treatment had greater mental health improvements (McAdams et al., 2001; Adler, 2012; Adler et al., 2015; Granic et al., 2020). Relatedly, hope and optimism are negatively associated with depressive symptoms (Alarcon et al., 2013) and Irving et al. (2004) showed that increases in hope positively predicted symptom improvement early in treatment.

Importantly, media are used by adolescents in their identity construction (Arnett, 1995) and strong narratives in video games can provide players with meaning and insight (Oliver et al., 2016). Although research is limited, media may increase hope through ‘underdog’ narratives (i.e., a character’s struggle toward a nearly unachievable goal; Prestin, 2013). The ‘underdog’ narrative may also promote hope in Journey players, who tumble from an idyllic desert into an eerie underground area filled with monsters. Situations like these can create a struggle for agency in players, being small, vulnerable, and having a limited range of action options. It may be exactly these challenging and limiting circumstances that make each choice feel meaningful (e.g., putting yourself at risk to save a co-player), allowing players to reflect on the meaningful agency in their own life.

As players continue playing Journey, each struggle is overcome and followed by brighter, more positive areas. The clear sequence of highs and lows may encourage players to find redemptive meaning in the game events. Through repetition players may transfer such a narrative arc to their own life’s story. Importantly, the use of redemptive sequences to provide meaning to past experiences has been associated with more optimism in emerging adults (McLean and Pratt, 2006).

Finally, coping strategies and emotion regulation play an essential role in the onset and maintenance of depressive symptoms (Nolen-Hoeksema, 1991; Silk et al., 2003; Aldao et al., 2010; Evans et al., 2015; Lo et al., 2017). Specifically, rumination has been repeatedly linked to the onset, maintenance, and recurrence of depression (Nolen-Hoeksema, 1991; Davis and Nolen-Hoeksema, 2000; Huffziger et al., 2009; Aldao et al., 2010; Lo et al., 2017). In contrast, a combination of distraction and problem solving seems to be associated with lower occurrence of depressive symptoms (Abela et al., 2007; Hilt et al., 2010).

Interestingly, a recent literature review concluded that commercial video games may be particularly well suited to improve emotion regulation (Villani et al., 2018), foster positive mood (e.g., Ryan et al., 2006; Russoniello et al., 2009; Osmanovic and Pecchioni, 2016; Reer and Krämer, 2018), and are used by youth to regulate their mood (Olson, 2010; Ferguson and Olson, 2013). Specifically, playing games is commonly linked to distraction and experimental research shows that more distracting video games indeed have beneficial effects on mood (Bowman and Tamborini, 2012). Journey is expected to similarly promote distraction among youth as it offers a relatively short, engaging, and rewarding play experience. Furthermore, initial evidence also relates playing strategic games to an increase in problem-solving skills (Adachi and Willoughby, 2017). While not a strategy game, negative emotional scenes in Journey require players to persevere despite negative emotions and overcome confrontations with monsters and other obstacles. We therefore hypothesize that problem solving matches an effective play style in Journey and rumination is discouraged.

A randomized controlled trial was conducted to test the effects and potential action mechanisms of Journey as an indicated depression prevention approach compared to another commercial game — Flower — and a passive control group. Comparing Journey to both Flower and a passive control group allowed us to distinguish non-specific effects of commercial video games and specific action mechanisms in Journey on depressive symptoms. Made by the same game studio, Journey and Flower are games with similar design and aesthetic. However, where Flower is a relaxing single-player game in which the player transforms their environment unopposed in a number of unrelated levels, Journey includes a narrative arc, is designed to evoke a range of both positive and negative emotions, and is a social, cooperative game. These specific design features of Journey (which are not present in Flower), correspond exactly to the action mechanisms we hypothesized prevent the exacerbation of depressive symptoms.

Participants were youth with elevated depressive symptoms aged 15 to 20 years old, as we expected the abstract nature of Journey to be more suited to this age range. We expected that youth who played Journey would show lower levels of depressive symptoms during post-test and six and 12 month follow-up, compared to the passive control group. In comparison, after playing Flower, participants were expected to experience less depressive symptoms than participants in the passive control group due to non-specific effects of game play, yet more depressive symptoms than participants playing Journey.

In regard to the action mechanisms, we hypothesized that after playing Journey participants would report feeling less rejection sensitivity, identify more redemptive sequences and agency in their narrative identity, have more hope and optimism, use less rumination, and use more problem solving and distraction. Moreover, we expected changes in these action mechanisms to mediate the effects of Journey compared to the active and passive control groups. Additionally, if conditions showed equal effects on depressive symptoms, exploring whether the action mechanisms explained variability in depressive symptoms over time would be illuminating. In that case, moderation analyses would allow us to examine whether action mechanisms moderated the effect of the conditions. Furthermore, exploring Journey’s immersive environment can promote the experience of intrinsic motivation, autonomy, competence, and flow thereby potentially promoting beneficial effects on depressive symptoms. Therefore, we tested whether a positive experience with Journey or Flower (i.e., experienced intrinsic motivation, autonomy, competence, and flow) moderated the effects of the games on depressive symptoms.

## Materials and Methods

### Participants

Participants were 244 adolescents and young adults with a mean age of 17.11 years (*SD* = 1.76). Two-thirds of participants were female (66.4%) and the vast majority was born in the Netherlands (93.4%). Overall participants’ education level was high, as only 9.1% of participants received vocational education, 32.1% received higher vocational education, and 58.8% received (pre-) university education. Finally, participants were quite positive about video games (*M* = 5.31, *SD* = 1.44 on a 7-point scale; 1 = *Do not like them at all*, 4 = *Like them somewhat*, 7 = *Like them very much*) and most played games at least once a week (71.7%) with only a few participants indicating not playing video games at all (3.7%).

### Procedure

**FIGURE 2:**
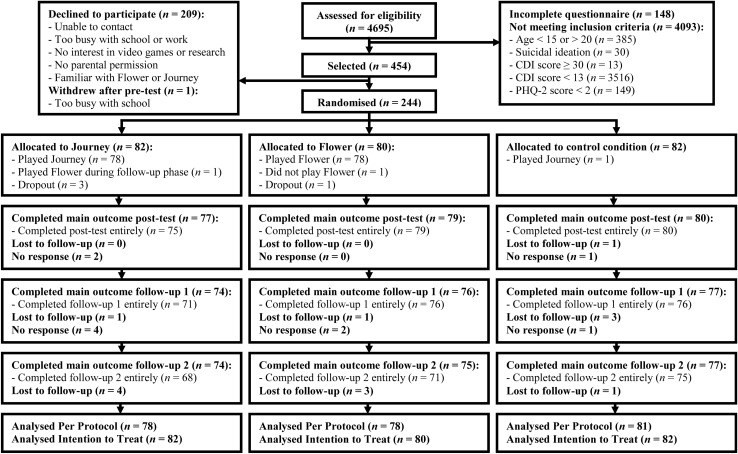
Flow chart participants from screening to 12 month follow-up.

Between November 2014 and June 2016 4,695 youth were screened for elevated depressive symptoms using a 10-min questionnaire (t0; see Figure 2 for the participant flow chart). Youth under the age of 18 were recruited through schools which had agreed to participate following an information letter and visit (17 secondary schools and one vocational tertiary school). Additionally, older youth were also recruited through flyers, social media posts, and the research participation system of the Radboud University. All youth and parents of secondary school students received written information regarding the study. Inclusion criteria and hypotheses were not communicated until the end of the study to reduce the potential for stigmatization and socially desirable answers. Parents provided passive consent for screening and youth were free to stop at any point.

We aimed for a strict inclusion of high-risk youth between the ages of 15 and 20 who were unfamiliar with Journey or Flower. Youth were invited for participation if they: (a) indicated elevated depressive symptoms on the Children’s Depression Inventory (CDI ≥ 13; Kovacs, 2001) and (b) indicated experiencing at least one of the core symptoms of a depressive disorder — depressed mood and anhedonia — the majority of the time over the past 2 weeks or alternatively experiencing both of the core symptoms on several days using the Patient Health Questionnaire 2 (Patient Health Questionnaire 2 ≥ 2; α = 0.54; Kroenke et al., 2003; Richardson et al., 2010)^2^. Youth were excluded at screening (*n* = 50) if they reported extremely severe depressive symptoms (CDI ≥ 30) and/or suicidal ideation (CDI item 9 = 2) as they were deemed in need of clinical assessment and/or intervention. These youth and youth scoring similarly during participation (*n* = 19) were contacted to discuss options for mental health services and parents of adolescent participants were involved if deemed appropriate. Medication use and receiving therapy were not exclusion criteria.

To detect a small effect size (*f* = 0.10) with a power of 0.90 while accounting for 10% attrition (correlation among repeated measures = 0.40; non-sphericity correction ε = 0.83; based on data from Poppelaars et al., 2016) 290 participants were required (GPOWER 3.9.1.2; Faul et al., 2007). Less youth met inclusion criteria and more declined participation than expected, therefore the original upper age limit was changed from 18 to 20 and the recruitment period was almost doubled. In the end, 454 youth were selected to participate and 244 participants were randomized. Selected youth often cited a busy schedule, no interest in scientific research or video games when declining participation (see Figure 2 for further information). Participants were more highly educated and liked video games more than youth who declined participation, while the two groups were similar otherwise (see Table 1).

**TABLE 1:** Descriptives (Means and Standard Deviations or Percentages) and χ^2^ or *F*-values of demographic variables for participating and non-participating youth separately.

		Participants		Non-participating youth		χ^2^ (1, *n* = 454)	*t* (452)
		*M*/%	*(SD)*	*M*/%	*(SD)*		
Age		17.11	(1.76)	16.97	(1.64)		0.92^*a*^
Gender						0.46	
	Female	66.4%		63.3%			
	Male	33.6%		36.7%			
Education level						26.03^b***^	
	Vocational	9.1%		21.4%			
	Higher vocational	32.1%		41.9%			
	(Pre-) University	58.8%		36.7%			
Born in the Netherlands						1.36	
	Yes	93.4%		90.5%			
	No	6.6%		9.5%			
Frequency of video game play						9.33^*c*^	
	Never	3.7%		6.2%			
	Once a month	8.2%		15.7%			
	Multiple times a month	16.4%		16.7%			
	Every week	21.3%		20.5%			
	Multiple times a week	26.6%		22.9%			
	Every day	23.8%		18.1%			
Liking video games		5.31	(1.44)	4.85	(1.67)		3.12^d**^
Depressive symptoms		17.27	(3.75)	17.63	(3.98)		−0.97
Core depressive symptoms		2.78	(1.02)	2.75	(0.97)		0.37

Significant effects are printed in bold. ^a^df = 442.28. ^b^df = 2, n = 453. ^c^df = 5, n = 454. ^d^df = 416.01. **p < 0.01, ***p < 0.001.

Participants filled in the 45-min pre-test questionnaire (t1) online after they and, if applicable, their parents had provided informed consent. At pre-test, depressive symptoms and all action mechanisms were assessed (i.e., rejection sensitivity, narrative identity, hope and optimism, and coping strategies; see Instruments). Additionally, in order to ensure participants on average had similar expectations regarding potential benefits of playing Journey and the control game Flower, all participants were shown trailers for Journey and Flower with similar messages at pre-test (see Boot et al., 2013 for the importance of equal expectations in active control conditions). Expectations regarding well-being were checked using self-developed items (based on Scholten et al., 2016) on a 7-point scale (1 = *Not at all* to 7 = *Definitely*; Flower: α = 0.82 and Journey: α = 0.83).

Next, an independent researcher randomized participants to a passive condition (*n* = 82) or to play either Journey (*n* = 82) or Flower (*n* = 80). Initially, randomization was done once a month stratified by gender. After the age range was adjusted, blocked randomization lists with a random block size order (blocks of three, six or nine participants) were created separate for gender and school or other recruitment.

Participants in the game conditions were given a logbook and a PlayStation 3 console pre-installed with their assigned game for 4 weeks. They were requested to complete the game at least once, but no further restrictions were given to encourage naturalistic gameplay. Flower takes 2 to 3 h to complete compared to approximately 3 h for Journey. Meanwhile, passive control participants continued their normal routine.

Within 2 weeks after the intervention period, participants filled in an online post-test questionnaire (t2) which was repeated six (t3) and 12 months (t4) later. All questionnaires repeated the assessment of depressive symptoms and the action mechanisms. For participants who played Journey or Flower game engagement measures were included at post-test (see Instruments). Outcomes that are less theoretically relevant to this paper are not reported, however an overview of all measures is provided in the Supplementary Materials (see Supplementary Table 1) and the full data is available at the DANS EASY online data repository (Poppelaars et al., 2020). Moreover, all questionnaires included a number of filler items (e.g., items regarding academic achievements) to distract from the study’s purpose. Questionnaires without an official Dutch translation were translated into Dutch separately by two researchers and back translated by a third researcher. Any discrepancies were solved through discussion.

In total, 217 participants (88.9%) completed all five assessments of depressive symptoms. This resulted in a power of 0.77 to detect small effects (*f* = 0.10) and a power of 0.99 to detect small to medium effects (*f* = 0.15; ANOVA repeated measures and within-between interaction; correlation among repeated measures = 0.36; non-sphericity correction ε = 0.77; GPOWER 3.9.1.2; Faul et al., 2007).

Participants received course credits, monetary rewards (€35,- in gift certificates) or a combination as compensation for the questionnaires. After the last questionnaire, passive control participants were offered Flower and/or Journey. Ethical approval for the study was granted by the ethical committee of the Faculty of Social Sciences at Radboud University ECSW2014-1003-201) and the trial was pre-registered at the Dutch Trial Register (Nederlands Trial Register: NL4873).

### Control Game

**FIGURE 3:**
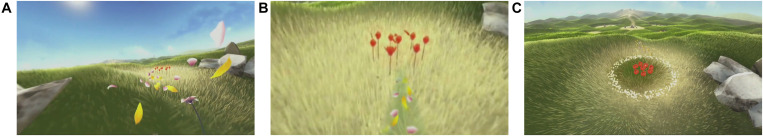
Gameplay in Flower. Players steer flower petals toward **(A)** and past flowers **(B)** which then bloom and transform the environment **(C)**. These images are reproduced from Flower by Thatgamecompany (2009b) with the permission of the copyright holder Sony Interactive Entertainment.

Similar to Journey, Flower was designed by Thatgamecompany (2009b) and combines minimalistic game controls with powerful game mechanics. Both games have pleasing aesthetics and immersive music and are appealing to frequent and non-frequent players. In Flower players explore six levels or landscapes controlling the wind on which a growing string of flower petals float (see Figure 3)^3^. With each flower that the player’s petals touch more flowers bloom and the landscape becomes more vibrant. Although players cannot die, in the penultimate level they lose some petals if they fly too close to electricity poles. Critics and the majority of players received Flower positively (Metacritic, 2009; Thatgamecompany, 2009a).

### Instruments

#### Depressive Symptoms

Severity of depressive symptoms was measured with the CDI (Kovacs, 2001) using 27 items consisting of three related statements. Participants indicate the statement reflecting their experience in the past 2 weeks (e.g., 0 = *I get sad from time to time*, 1 = *I get sad often*, 2 = *I’m always sad*). To account for the extended age range of the study, slight adaptations made items suitable for all participants (e.g., “I’m as good as other children.” became “I’m as good as other youth.”). A sum score is calculated ranging from 0 to 54 with 13 reverse scored items; higher scores indicate more severe depressive symptoms. Reliability was acceptable to good (α = 0.75–0.86).

#### Action Mechanisms

##### Rejection sensitivity

The tendency to anxiously expect rejection was measured using the eight item Rejection Sensitivity Questionnaire (Downey and Feldman, 1996). Participants were presented with possible rejection situations (e.g., “You ask a friend to do you a big favor.”) and asked how concerned they would be over the reaction on a 6-point scale (1 = *very unconcerned* to 6 = *very concerned*; rejection concern) and to what extent they would expect a positive reaction on a 6-point scale (1 = *very unlikely* to 6 = *very likely*; acceptance expectancy). For each item the rejection concern score is multiplied by the reverse score of acceptance expectancy. A mean score with a possible range of 1 to 36 is calculated with higher scores indicating more rejection sensitivity. Reliability was acceptable to good (α = 0.73–0.80).

##### Narrative identity

Participants wrote approximately five lines each on a high, low, and turning point in their life based on the Life Story Interview (McAdams, 2008). Three graduate students trained by the first and second author coded redemptive sequences and agency (McAdams, 1999, 2002). Coders could score one point for agency for: (a) gaining insight or control in life, (b) gaining status or victory, (c) attaining an achievement or taking responsibility, and (d) feeling empowered by an authority figure. A redemptive sequence was coded (0 = *no*, 1 = *yes*) when a clearly negative state resulted in a positive state or outcome. An extra point per category was scored for additional positive outcomes for: (a) agency, (b) social relationships, or (c) spiritual experience. Sum scores for redemptive sequences and agency were calculated from the three prompts with a potential range of 0 to 12. In total 819 sets of narrative fragments were coded of which 128 (15.6%) were coded independently by two coders. Intraclass Correlation Coefficients based on a single rating, absolute agreement, 1-way random effects model showed poor to moderate interrater reliability (Intraclass Correlation Coefficients = 0.58, 95% CI [0.45,0.68]) for agency and moderate to good reliability (Intraclass Correlation Coefficients = 0.67, 95% CI [0.56,0.75]) for redemptive sequences (Koo and Li, 2016). Complete agency and redemption data were available for 161 (66.0%) and 160 (65.6%) participants, respectively.

##### Hope and optimism

Hope and optimism were measured using the Global Positive Expectancies scale (Carvajal, 2012). A mean score is calculated from the eight items (e.g., “I always look on the bright side of things.”) measured on a 4-point scale (1 = *None of the time*, 2 = *Some of the time*, 3 = *Most of the time*, 4 = *All of the time*). Reliability was acceptable to good (α = 0.75–0.84).

##### Coping strategies

Participants’ response to depressed moods was measured using the Children’s Response Styles Questionnaire (Abela et al., 2007). Specifically, 13 items measured rumination (e.g., “When I am sad, I think about how sad I feel.”) and eight items measured distraction and problem solving (e.g., “When I am sad, I think of a way to make my problem better.”) on a 4-point scale (0 = *Almost never*, 1 = *Sometimes*, 2 = *Often*, 3 = *Almost always*). Reliability was good for the Rumination subscale (α = 0.85–0.88), however reliability for the Distraction and Problem-Solving subscale was questionable to acceptable (α = 0.67–0.74).

#### Game Engagement

##### Intrinsic motivation

The Interest/Enjoyment subscale of the Intrinsic Motivation Inventory (Ryan, 1982; McAuley et al., 1989) assessed intrinsic motivation experienced during gameplay with excellent reliability (α = 0.95). The seven items (e.g., “I would describe this game as very interesting.”) were assessed on a 7-point scale (1 = *Not at all true*, 4 = *Somewhat true*, 7 = *Very true*) at post-test. A mean score was calculated after reverse scoring two items, where higher scores indicated more motivation.

##### Autonomy and competence

The Player Experience of Need Satisfaction questionnaire (Ryan et al., 2006; Immersyve, 2007) measures the experience of autonomy (e.g., “I experienced a lot of freedom in the game.”) and competence (e.g., “I feel very capable and effective when playing.”). A mean score was calculated for each three-item subscale measured on a 7-point scale (1 = *Strongly disagree* to 7 = *Strongly agree*) with good reliability (Autonomy: α = 0.84 and Competence: α = 0.88).

##### Flow

Participants were given a description of flow and the way people experience it (Novak et al., 2000). Next, participants rated on 9-point scales if they had ever experienced flow (1 = *Not at all sure* to 9 = C*ompletely sure*), how frequently they had experienced flow (1 = *Never* to 9 = *Very frequently*), and if most of the time they had experienced flow (1 = *Strongly disagree* to 9 = *Strongly agree*) while playing Journey or Flower. Higher mean scores indicate a stronger experience of flow during gameplay with excellent reliability (α = 0.91).

### Statistical Analyses

To start, the descriptive statistics reported below include all available data (we checked that excluding participants who did not follow protocol or who dropped out did not change the significance of any reported descriptive statistics). Randomization success was checked using one-way ANOVAs and chi-square tests on demographic variables. Next, both depressive symptoms and action mechanisms at each time point and game engagement variables were compared between conditions using one-way ANOVAs. Furthermore, correlations between age, depressive symptoms, and action mechanisms at pre-test and the game engagement variables were examined. As the age range in our study was extended and many of our dependent variables showed significant correlations with age, we used age as a covariate in all further analyses.

Mixed Effects Models were used for both the intention to treat and per protocol analyses. Mixed effects models is a recommended method to analyze longitudinal data with missing data points and can model the covariance structure of participants’ repeated measurements over time (Gueorguieva and Krystal, 2004; Krueger and Tian, 2004), while the interpretation is similar to commonly used Repeated Measures ANOVAs (Littell et al., 2000). Using Residual Maximum Likelihood the best fitting solution is estimated based on all available data, including participants missing one or more of the repeated assessments. Moreover, rather than assuming sphericity for repeated measurements, mixed effects models allow for more appropriate modeling of the correlations between repeated assessments from subjects through the covariance matrix (Gueorguieva and Krystal, 2004). For each dependent variable the best fitting covariance matrix was selected based on the Akaike information criterion and the Schwarz Bayesian criterion (following Littell et al., 2000). A selection was made from 7 common covariance matrices: (a) Unstructured, which uses the covariance and variance values from the sample; (b) Compound Symmetry, which specifies equal covariance between repeated measures; (c) Autoregressive; and (d) Toeplitz, which both specify that covariance between repeated measures decrease with more time between assessments in specific ways; (e) Compound Symmetry Heterogeneous; (f) Autoregressive Heterogeneous; and (g) Toeplitz Heterogeneous, the last three of which allow variances to differ between repeated measures compared to their counterparts (see Littell et al., 2000).

Next, the effect of condition on depressive symptoms and the action mechanisms over time was tested. Mixed effects models used all available time-points for depressive symptoms (t0-t4) and each action mechanism (t1-t4), entering time and condition as categorical variables and age as a continuous covariate. In order to use age as a covariate, the variable was mean centered. The model included fixed effects for time, age, condition, Time × Age, and Time × Condition.

Further, mediation of the action mechanisms could not be tested as the effect of condition on change in depressive symptoms was not significant. Therefore, we instead tested for moderation effects of the action mechanisms. Separate mixed effects models were used to test whether change in the action mechanisms as well as game engagement moderated the effect of condition on depressive symptoms over time. To this end, the original mixed effects model for depressive symptoms was extended by adding the centered action mechanism change variable or centered game engagement variable as a continuous covariate. Additionally, the interactions of the action mechanism or game engagement variable with condition, time, and Time × Condition were included as fixed effects. For all action mechanisms, centered change variables were created by subtracting pre-test scores from the final follow-up scores and mean centering these scores. Moderation analyses only included data from participants with a valid centered action mechanism change variable or centered game engagement variable.

The per protocol analyses included only participants who followed protocol and played Journey or Flower or neither game as prescribed by the randomization. These analyses were only reported if they deviated from the intention to treat analyses. To reduce the possibility of chance findings α = 0.01 was used for the moderation analyses. All analyses were performed in SPSS 25 (Ibm Corp, 2017) and Bonferroni corrections were applied to all *post hoc* tests.

## Results

### Descriptive Statistics

Randomization was successful as there were no significant differences between conditions on the demographic variables, expectations of Journey, expectations of Flower, and on receiving other treatment during the study period (see Table 2). In addition, a RM-ANOVA of expectations showed participants had similar expectations of the potential effect of Journey and Flower at pre-test (*F*(1, 241) < 0.01, *p* = 0.97, η*^2^_*p*_* < 0.01) and this was not moderated by the condition to which participants were randomized (*F*(2, 241) = 0.05, *p* = 0.95, η*^2^_*p*_* < 0.01). Participants in both game conditions on average recorded approximately three sessions of gameplay in their logbooks (Flower *M* = 3.03, *SD* = 1.76; Journey *M* = 3.25, *SD* = 1.93; *t(148)* = 0.73, *p* = 0.47), but Journey on average was played longer than Flower (Flower *M* = 2 h 36 min, *SD* = 1 h 55 min; Journey *M* = 3 h 20 min, *SD* = 2 h 16 min; *t (145)* = 2.09, *p* < 0.05). Additionally, on average playing Journey was experienced as more engaging than playing Flower, as participants playing Journey on average reported experiencing more intrinsic motivation (Flower *M* = 3.66, *SD* = 1.54; Journey *M* = 4.73, *SD* = 1.53; *F*(1, 151) = 18.40, *p* < 0.001), autonomy (Flower *M* = 3.40, *SD* = 1.52; Journey *M* = 4.48, *SD* = 1.49; *F*(1, 151) = 19.85, *p* < 0.001), competence (Flower *M* = 3.71, *SD* = 1.60; Journey *M* = 4.67, *SD* = 1.37; *F*(1, 151) = 15.59, *p* < 0.001), and flow (Flower *M* = 3.94, *SD* = 2.10; Journey *M* = 4.96, *SD* = 2.20; *F*(1, 151) = 8.60, *p* < 0.01) than participants playing Flower. Further, no significant differences were found on depressive symptoms or any of the action mechanisms between conditions on their first assessment nor on any other time point (see Supplementary Table 2).

**TABLE 2:** Descriptives (Means and Standard Deviations or Percentages) per condition and χ^2^ or *F*-values for demographic variables at T0, intervention expectation at T1, and other treatment during the study.

		Total *M (SD)*/%	Journey *M (SD)*/%	Flower *M (SD)*/%	Passive control *M (SD)*/%	χ^2^	*F*(2, 241)
Age		17.11	17.13	17.16	17.05		0.09
		(1.76)	(1.81)	(1.79)	(1.71)		
Gender						0.07^*a*^	
	Female	66.4%	65.9%	67.5%	65.9%		
	Male	33.6%	34.1%	32.5%	34.1%		
Education level						5.87^*b*^	
	Vocational	9.1%	6.1%	10.1%	11.0%		
	Higher vocational	32.1%	32.9%	24.1%	39.0%		
	(Pre-) University	58.8%	61.0%	65.8%	50.0%		
Born in the Netherlands						0.87^*a*^	
	Yes	93.4%	93.9%	95.0%	91.5%		
	No	6.6%	6.1%	5.0%	8.5%		
Frequency of video game play						10.30^*c*^	
	Never	3.7%	2.4%	3.8%	4.9%		
	Once a month	8.2%	11.0%	8.8%	4.9%		
	Multiple times a month	16.4%	15.9%	21.3%	12.2%		
	Every week	21.3%	22.0%	23.8%	18.3%		
	Multiple times a week	26.6%	24.4%	18.8%	36.6%		
	Every day	23.8%	24.4%	23.8%	23.2%		
Liking video games		5.31	5.35	5.20	5.37		0.33
		(1.44)	(1.43)	(1.53)	(1.37)		
Expectation							
	Flower	4.28	4.22	4.33	4.30		0.19
		(1.22)	(1.29)	(1.06)	(1.31)		
	Journey	4.28	4.20	4.33	4.32		0.30
		(1.23)	(1.30)	(1.16)	(1.24)		
Other treatment						1.13^*d*^	
	No	58.9%	57.5%	63.9%	55.7%		
	Yes	41.1%	42.5%	36.1%	44.3%		
Core depressive		2.78	2.78	2.78	2.79		
symptoms at screening		(1.02)	(1.02)	(0.94)	(1.11)		0.01

*^a^χ^2^ (2, n = 244). ^b^χ^2^ (4, n = 243). ^c^χ^2^ (10, n = 244). ^d^χ^2^ (2, n = 224).*

Correlations between age, depressive symptoms, and action mechanisms at pre-test and the game engagement variables are provided in Table 3. Notably, age correlated significantly with several dependent variables, showing that older participants on average showed more depressive symptoms, more rejection sensitivity, more agency in their identity narratives, less hope and optimism, and more rumination than younger participants. Furthermore, it stands out that depressive symptoms were not found to be related to agency in identity narratives or distraction and problem solving in this sample.

**TABLE 3:** Correlations between age, depressive symptoms, and action mechanisms at pre-test and game engagement variables.

	Age	DS	RS	NR	NA	H&O	RUM	D&P	IM	AT	CM
Age											
DS	0.26***										
RS	0.16*	0.47***									
NR	−0.01	−0.15*	−0.04								
NA	0.18**	0.06	0.13*	0.40***							
H&O	−0.28***	−0.63***	−0.44***	0.08	−0.01						
RUM	0.18**	0.39***	0.34***	0.02	0.11	−0.29***					
D&P	0.04	−0.10	−0.16*	0.18**	0.15*	0.26***	−0.02				
IM	−0.03	0.08	0.14	−0.15	−0.02	0.01	0.07	0.08			
AT	−0.04	< 0.01	0.14	−0.04	< −0.01	0.12	0.02	0.14	0.80***		
CM	0.08	−0.05	0.11	−0.03	0.07	0.10	0.07	0.18*	0.67***	0.66***	
FL	−0.01	< 0.01	0.03	−0.08	0.05	0.18*	< −0.01	0.07	0.72***	0.64***	0.59***

Significant effects are printed in bold. DS, Depressive Symptoms; RS, Rejection Sensitivity; NR, Narrative Redemptive Sequences; NA, Narrative Agency; H&O, Hope and Optimism; RUM, Rumination; D&P, Distraction and Problem Solving; IM, Intrinsic Motivation; AT, Autonomy; CM, Competence; FL, Flow. *p < 0.01, **p < 0.01, ***p < 0.001.

### Depressive Symptoms

**FIGURE 4:**
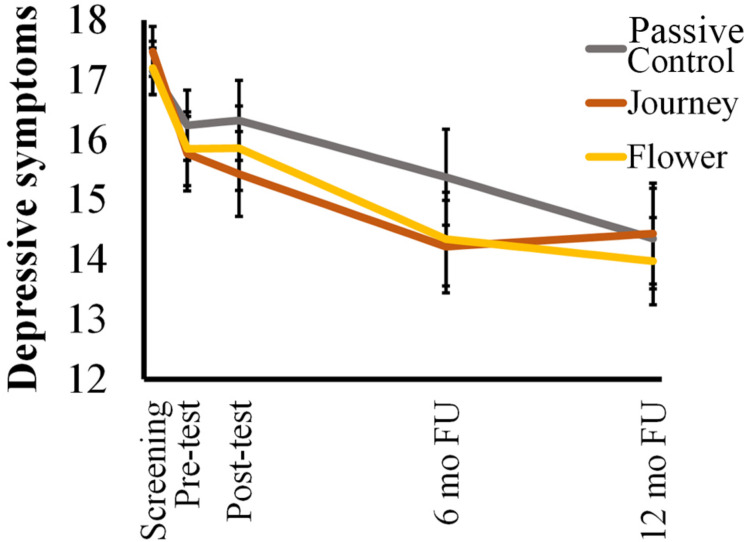
Trajectories of mean depressive symptoms (CDI) scores by condition. Error bars represent standard errors.

Next, a mixed effects model was used to test if condition was associated with differences in depressive symptoms over time. First, the best fitting covariance matrix was determined to be Toeplitz with heterogeneous variances as indicated by the second lowest Akaike’s Information Criterion (6819.13) and the lowest Schwarz’s Bayesian Criterion (6864.62; following recommendations of Littell et al., 2000). While a significant main effect of time (*F*(4, 295.52) = 16.44, *p* < 0.001) and a significant Time × Age interaction (*F*(4, 296.29) = 7.85, *p* < 0.001) were found, no Time × Condition interaction was found (*F*(8, 295.55) = 0.85, *p* = 0.56; see Figure 4). Thus, results indicate that participants in all conditions showed a similar decrease in depressive symptoms over the study period (t0-t4: β = −2.78, *SE* = 0.75, 95% CI [−4.26, −1.30]).

When comparing screening and the final follow-up, age did not influence the decrease in symptoms (t0-t4: β = −0.03, *SE* = 0.25, 95% CI [−0.52, 0.46]). Rather, *post hoc* analyses indicated that age had an effect on the timing of the change in depressive symptoms. Younger participants showed an early decrease in depressive symptoms between screening and pre-test (15-year olds *p* < 0.001; 16-year olds *p* < 0.001; 17-year olds *p* < 0.001) that was not found for older participants. A significant decrease in symptoms later on in the study was only seen in participants aged 17 years and older (17-year olds t2-t3 *p* < 0.01 18-year olds t2-t3 *p* < 0.01; 19-year olds t2-t3 *p* < 0.05; 20-year olds t2-t4 *p* < 0.001). Thus, younger participants decreased in depressive symptoms earlier but not more compared to older participants. Moreover, despite the overall decrease in depressive symptoms, approximately 50.2% of the participants still met the inclusion criteria for elevated depressive symptoms at the final follow-up with no differences between conditions (χ*2* (2, *n* = 223) = 0.47, *p* = 0.79).

### Action Mechanisms

The results of the mixed effects models for all action mechanisms are reported in Table 4. Main effects of time were found for (a) rejection sensitivity and (b) hope and optimism. Time × Age and Time × Condition interactions were not found for either variable. Findings indicate that participants in all conditions and of all ages showed a mean decrease in rejection sensitivity (t1-t4: β = −1.11, *SE* = 0.46, 95% CI [−2.01, −0.21]) and mean increase in hope and optimism (t1-t4: β = 0.13, *SE* = 0.06, 95% CI [0.02, 0.24]) during the study. The mixed effects models of all other action mechanisms showed no significant effects of time, Time × Age, and Time × Condition. This indicates that narrative redemptive sequences, narrative agency, rumination, and distraction and problem solving remained stable for all conditions and all ages during the study period.

**TABLE 4:** The effect of condition on action mechanisms over time using mixed effects models.

	Covariance matrix	Fit indices		Time		Time × Age		Time × Condition	
		AIC	BIC	*df*	*F*	*df*	*F*	*df*	*F*
RS	TP	4896.52	4915.78	3, 394.78	5.65***	3, 394.69	1.16	6, 394.65	0.85
NR^*a*^	CS	2696.81	2706.19	3, 596.94	2.45	3, 595.90	0.05	6, 596.57	0.61
NA^*a*^	CS	2376.05	2385.43	3, 601.33	0.98	3, 600.27	0.49	6, 600.94	0.34
H&O	TP	949.02	968.29	3, 381.33	5.90***	3, 380.64	1.51	6, 381.09	0.90
RUM	TP	5903.23	5922.49	3, 397.57	1.21	3, 396.96	0.03	6, 397.48	1.65
D&P	TP	4873.53	4892.79	3, 417.53	1.77	3, 415.56	1.78	6, 417.47	0.35

Significant effects are printed in bold. AIC, Akaike Information Criterion; BIC, Schwarz Bayesian Criterion; RS, Rejection Sensitivity; NR, Narrative Redemptive Sequences; NA, Narrative Agency; H&O, Hope and Optimism; RUM, Rumination; D&P, Distraction and Problem Solving; TP, Toeplitz; CS, Compound Symmetry. ^a^ Excluding 7 participants without any codeable narrative data. ***p < 0.001.

### Moderation of Action Mechanisms

As there was no effect of condition on change in depressive symptoms, no mediation analyses could be performed. Therefore, we tested whether change in the action mechanisms moderated the effect of condition on depressive symptoms. Mixed effects model results are reported in Table 5 and show that all moderation analyses replicated the main effect of time and the Time × Age interaction on depressive symptoms. The Time × Age interaction can be interpreted the same way as in the main outcome. However, when hope and optimism was added as a moderator we additionally saw a significant decrease in depressive symptoms for the 16-year olds later in the study (t1-t3 *p* < 0.01).

**TABLE 5:** The effect of condition on depressive symptoms over time moderated by action mechanisms or game engagement using mixed effects models.

	Fit indices		Time		Time × Age		Time × Δ AM/GE		Time × Condition		Time × Δ AM/GE × Condition	
	AIC	BIC	*df*	*F*	*df*	*F*	*df*	*F*	*df*	*F*	*df*	*F*
RS	6345.68	6390.41	4, 278.17	16.13***	4, 278.84	6.24***	4, 278.14	9.87***	8, 278.17	0.69	8, 278.23	1.52
NR	5384.50	5427.72	4, 230.97	13.70***	4, 231.67	5.32***	4, 229.74	1.94	8, 231.00	0.54	8, 229.96	0.94
NA	5401.58	5444.86	4, 231.27	13.59***	4, 231.94	5.22***	4, 230.15	0.77	8, 231.29	0.65	8, 230.34	1.16
H&O	6254.26	6299.04	4, 290.39	20.42***	4, 290.85	5.89***	4, 289.70	32.97***	8, 290.40	0.94	8, 289.81	0.36
RUM^*a*^	6305.04	6349.73	4, 280.13	15.25***	4, 280.90	6.25***	4, 280.18	12.57***	8, 280.14	0.77	8, 280.22	1.97
D&P^*b*^	6332.73	6377.43	4, 276.18	15.63***	4, 276.78	6.04***	4, 275.81	5.19***	8, 276.18	0.72	8, 275.80	0.43
IM	4305.96	4347.22	4, 194.73	10.60***	4, 195.11	5.86***	4, 194.00	0.45	4, 194.73	0.26	4, 193.96	1.23
AT	4300.89	4342.15	4, 194.30	9.01***	4, 194.91	6.40***	4, 194.35	0.34	4, 194.26	0.23	4, 194.36	2.86
CM	4304.21	4345.47	4, 193.76	10.08***	4, 194.12	5.86***	4, 193.30	0.67	4, 193.75	0.26	4, 193.35	0.95
FL	4314.04	4355.30	4, 193.96	11.60***	4, 194.33	5.91***	4, 194.71	0.77	4, 193.92	0.25	4, 194.70	0.41

Significant effects are printed in bold. The covariance matrix Toeplitz with heterogeneous variances was used for all analyses. ^Δ^AM, Change in Action Mechanisms; GE, Game Engagement Variables; AIC, Akaike Information Criterion; BIC, Schwarz Bayesian Criterion; RS, Rejection Sensitivity; NR, Narrative Redemptive Sequences; NA, Narrative Agency; H&O, Hope and Optimism; RUM, Rumination; D&P, Distraction and Problem Solving; IM, Intrinsic Motivation; AT, Autonomy; CM, Competence; FL, Flow. ^a^The interaction Time × Change in Rumination × Condition was significant in the per protocol analysis, however with the exclusion of an extreme multivariate outlier the effect was not significant in both the per protocol and intention to treat analyses. ^b^The interaction Time × Change in Distraction and Problem Solving was not significant in the full sample, however with the exclusion of an extreme multivariate outlier the effect is significant. ***p < 0.001.

Further, results indicated that both change in narrative redemptive sequences and change in narrative agency did not moderate the effect of time or the interaction of Time × Condition on depressive symptoms. Indicating that change in narrative aspects had no effect on the decrease in depressive symptoms over time in the current study.

**FIGURE 5:**
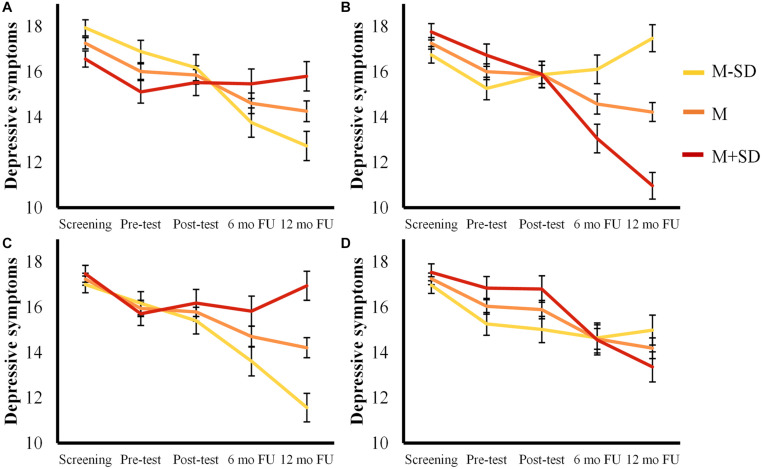
Estimated mean trajectories for depressive symptoms moderated by **(A)** Rejection Sensitivity, **(B)** Hope and Optimism, **(C)** Rumination and, **(D)** Distraction and Problem solving. Error bars represent standard errors.

On the other hand, change in (a) rejection sensitivity, (b) hope and optimism, (c) rumination, and (d) distraction and problem solving were found to moderate the change of depressive symptoms over time (see Figure 5). First, participants who decreased more in rejection sensitivity showed a larger decrease in depressive symptoms across the study (t0-t4: β = 0.68, *SE* = 0.19, 95% CI [0.31, 1.05]). Second, participants who showed a larger decrease in rumination also showed a larger decrease in depressive symptoms (t0-t4: β = 0.38, *SE* = 0.10, 95% CI [0.19, 0.58]). In the opposite direction, participants who showed a larger increase in hope and optimism, showed a larger decrease in depressive symptoms (t0-t4: β = −7.96, *SE* = 1.27, 95% CI [−10.48, −5.45]). Similarly, an increase in distraction and problem solving was related to a larger decrease in depressive symptoms (t0-t4: β = −0.45, *SE* = 0.18, 95% CI [−0.80, −0.10]). Importantly, none of these action mechanisms moderated the interaction of Time × Condition on depressive symptoms. This indicates that the associations between changes in action mechanisms and changes in depressive symptoms were not specific to any condition.

### Moderation of Game Engagement

Finally, we tested if the experience participants had playing Journey or Flower impacted their change in depressive symptoms or moderated the effect of condition on change in depressive symptoms (see Table 5). These analyses again confirmed the decrease in depressive symptoms over time and the age related differences in trajectories. The only discrepancy was that when competence was added as a moderator we saw the decrease in depressive symptoms for 17-year olds later in the study was no longer significant at α = 0.01 (t2-t3 *p* = 0.02). The same effect was seen for motivation (t1-t4 *p* = 0.02) and autonomy (t1-t4 *p* = 0.01) in the per protocol analyses. Importantly, none of the engagement variables moderated the effect of time or Time × Condition.

## Discussion

This study showed no beneficial effects of playing the commercial video game, Journey, on progression of depressive symptoms of adolescents and young adults with elevated depressive symptoms above and beyond an active and passive control. Nevertheless, youth in all conditions decreased in depressive symptoms, became less sensitive to rejection, and experienced more hope and optimism over the study period. Participants who, over time, experienced less rumination, less rejection sensitivity, more distraction and problem solving, and particularly more hope and optimism showed the strongest decrease in depressive symptoms. Thus, while no action mechanisms could be identified for Journey specifically, we confirmed that hope and optimism, rumination, rejection sensitivity, and distraction and problem solving are promising targets for future depression prevention efforts.

### Journey Effects

Contrary to our hypothesis youth assigned to play Journey in the present study did not improve more or faster in their depressive symptoms compared to youth in the other two conditions. Thus, anecdotal evidence of Journey players reporting mental health benefits did not translate to benefits for a larger sample of youth with elevated depressive symptoms. This may suggest that the anecdotal evidence was based on people misattributing their mental health improvement to Journey. Alternatively, it may be that only a very small number of individuals are capable of benefiting from Journey as it is and the game mechanics would need to be strengthened considerably before the game could be used as a viable intervention.

However, elements of this trial may have also limited the potential for Journey to be effective. Specifically, selecting an appealing game can encourage the experience of autonomy, which when experienced more during therapy has been related to better treatment outcomes (Zuroff et al., 2012). The random assignment used within this trial may thus have reduced potential benefits. Therefore, research designs that allow for autonomous game selection are needed. Specifically, researchers may preselect youth interested in playing a specific game and use a reversal baseline design with (somewhat) reversible proximal outcomes during iterative game design (Kazdin, 2016), a matched control group design for distal outcomes (Nguyen et al., 2007) or a qualitative design to gain insight into youth’s experiences with specific games or game mechanics (Ribbens and Poels, 2009).

Moreover, the relatively short intervention period may have also limited the chance of Journey to be effective. We aimed to provide a naturalistic gameplay experience — allowing participants to play in their homes at a time of their choosing — however, the 4 week intervention period was based on logistics and participant burden rather than prior knowledge of how often youth would play Journey or how much time was needed for Journey to be effective. While most participants reported completing the game, few participants played through the game more than once. Thus, we cannot exclude that Journey would have been effective at a higher dose. However, had we made participants play through Journey or Flower several times, the results would likely not have been relevant to how youth interact with these games outside of a research context. Still in hindsight, more information regarding the way Journey is played outside of a research context could have helped in determining an optimal intervention period.

Additionally, Journey did not have a more beneficial effect on any of our proposed action mechanisms than our active and passive control conditions. Given that Journey was not designed to promote the hypothesized action mechanisms, a single playthrough may not have provided enough practice with these mechanisms to change existing thought and behavior patterns. For example, we hypothesized that repeated exposure to ambiguous losses that could be interpreted as rejections followed by reconnection to another co-player would reduce rejection sensitivity. However, as a single playthrough may at most contain three or four interactions with a co-player that are long enough to form a bond, this is understandably not enough for youth to change an ingrained sensitivity to rejection. Had Journey been more accessible to a younger target audience with more malleable action mechanisms (e.g., by using a less abstract narrative), the limited dose of the action mechanisms may have resulted in stronger effects.

Furthermore, this study’s results may be an indication that for games to impact action mechanisms the mechanics in these games need to be specifically designed. Particular attention in the design process should be given to: (a) allowing for repeated practice, (b) allowing for reflection, and (c) not giving opposing messages. Given the example of rejection sensitivity, the mechanic of interrupted social interactions is still promising, yet may need to be built in such a way that the player is guaranteed multiple rejection experiences. Additionally, the mechanic may be strengthened by providing room to reflect on ambiguous rejection experiences being less personal and/or less catastrophic.

Reflection, cognitive elaboration, and meaning making may be particularly important when targeting overarching cognitive interpretations such as rejection sensitivity, hope, and narrative identity. Narrative, meaningful choices, and social connections in video games have been shown to be vital to meaningful video games (Rogers et al., 2017), yet may have very little effect if the player rushes past these experiences. Similarly, meaningful experiences in everyday life can become an intricate part of one’s narrative identity or be forgotten and have no effect. Further processing of experiences through reflection and discussion with important others is vital for the experiences’ impact (McLean et al., 2007). Therefore, stimulating reflection and providing room for reflection during or after the gameplay experience may enhance the effectiveness of game mechanics.

Furthermore, opposing messages may make knowledge, insight, and skills harder to learn. For example, while the opportunity to find agency in challenging circumstances is a theme in Journey, this message may be overshadowed by the superficial uncontrollability and helplessness of players toward the environment and monsters. Thus, in the design process messages need to be carefully considered. Finally, for certain mechanisms or target groups, the connection to daily life may have to be made more explicit. Although repeated exposure and in-game reflection may be enough for some to connect a redemptive arc to their own experiences, others will need more assistance in understanding the redemptive meaning of the story line and to talk about experiences from their own life that may fit that same structure.

### Trajectories of Depressive Symptoms and Action Mechanisms

Although Journey had no influence on depressive symptoms, overall, participating youth did show a significant decrease in depressive symptoms. Rather than decreasing symptoms, prevention is intended to avoid expected symptom increases and the onset of depressive disorders over time (Gillham et al., 2000). Thus, we may have failed to include participants most at risk to develop a depressive disorder, despite selecting on both elevated depressive symptoms and presence of core symptoms of depression. However, the observed decrease in symptoms may not have occurred outside of a research context and be related to study participation. Multiple studies have demonstrated that depressive symptoms decrease with repeated assessment, which may be due to measurement artifacts or possible therapeutic value of researcher attention, enhanced self-awareness or triggering of coping (Sharpe and Gilbert, 1998; Arrindell, 2001). Alternatively, the self-limiting nature of depressive symptoms may have resulted in an overall decrease in symptoms across the 14-month study. Indeed, a recent representative study of the Dutch adult population indicated that 79.0% of subclinically depressed and 73.6% of clinically depressed individuals recovered within 1 year (ten Have et al., 2017).

Depressive symptom trajectories in the current study may also be indicative of developmental patterns as significant decreases in symptoms occurred earlier for younger participants than for older participants. This suggests that younger participants’ elevated depressive symptoms are less stable and may decrease relatively quickly with minimal intervention. In contrast, young adults seem to have more persevering symptoms which is alarming as depressive symptoms in and of themselves are damaging (Wesselhoeft et al., 2013). And while symptoms had decreased by the end of the study, more intense interventions may be needed for young adults. Maintaining mechanisms of depressive symptoms, such as rejection sensitivity, may have become more ingrained making quick symptom decreases less likely. Indeed, previous research has indicated that decreases in depressive symptoms are common in late adolescents, while depressive symptoms are more stable in young adulthood (Yaroslavsky et al., 2013). Future research is needed to explore if prevention programs need to contain different elements for older youth or youth with consistently elevated depressive symptoms.

Similar to the improvement in depressive symptoms, participants across conditions had more positive expectations for their future and were less rejection sensitive at the end of the study. Again, repeated assessment may be a factor in these positive developments, as Arrindell (2001) shows that this effect is found in a range of well-being measures. Alternatively, participants may have truly become more hopeful and optimistic and less rejection sensitive due to general aspects of participating in research mentioned above. Additionally, hope and optimism may have increased as participants expected positive outcomes from the study.

### Action Mechanism Effects on Symptom Improvement

Although we could not examine the mediation of the action mechanisms, four of the action mechanisms moderated improvements in depressive symptoms. As Journey did not successfully change the action mechanisms, the direction of these effects could not be established in the current study. Further research using interventions that successfully improve hope and optimism, decrease rumination, decrease rejection sensitivity, and/or improve distraction and problem solving is needed to confirm that depressive symptom prevention is mediated by these action mechanisms. However, it is still promising that four of the action mechanisms influenced change in depressive symptoms. Most prominently, increases in hope and optimism were strongly related to decreasing depressive symptoms. Since underdog narratives have been shown to increase hope short-term (Prestin, 2013), future studies should explore this and other game mechanics likely to promote hope and optimism and examine long-term effects.

Additionally, using more adaptive coping — less rumination and more distraction and problem solving — was associated with less depressive symptoms in the current study. Consistent with earlier research rumination was more strongly related to depressive symptoms than distraction and problem solving (Aldao et al., 2010; Hilt et al., 2010). However, a recent review indicated that the choice of coping strategy is more problematic than the ability to perform adaptive coping strategies (Liu and Thompson, 2017). Therefore, applied games may be more effective if they can steer youth away from ruminative responses and toward more adaptive coping strategies (Liu and Thompson, 2017).

Furthermore, similar to previous research, this study showed that a decrease in rejection sensitivity was related to a decrease in depressive symptoms (Marston et al., 2010). These findings are in line with the social risk hypothesis of depression which posits that subclinical depressive symptoms are an adaptive response to the threat of social exclusion, directing attention to social cues and heightening sensitivity to rejection (Allen and Badcock, 2003). If more support is found for the social risk hypothesis, rejection sensitivity and the experience of social threat can be major targets for applied depression games given the social nature of video games (Entertainment Software Association, 2019) and their strong capacity to evoke rejection experiences (Tuijnman et al., 2017).

### Strengths, Limitations, and Future Directions

Despite not showing additional benefits from Journey on depressive symptom, this study has a number of valuable strengths and can serve as a source of important lessons for future studies. In terms of strengths the well powered randomized controlled trial design — the gold standard in prevention research — with an active and passive control condition allows us to draw clear conclusions from this study. Additionally, the way interventions were provided approximated naturalistic gameplay, making the outcomes relevant to the reality of commercial video game use. In future studies using a similar three-condition design can help distinguish specific and non-specific effects in effective prevention games. Another strength of this study is the use of an existing resource, a commercial video game, to study the depression prevention potential of less commonly investigated action mechanisms. Although the current study did not show beneficial effects of Journey, we could have had similar results at far greater costs with a newly developed traditional intervention or applied game.

Yet this study also has several limitations. First, approximately half of the screened youth meeting inclusion criteria decided not to participate. Participating youth liked video games more and were more highly educated, which indicates self-selection. Although youth who find games less appealing may not be the main target audience of video game interventions, Journey could have impacted these youth more as they have less previous exposure to games. Thus, our recruitment strategies may have skewed the results. Recruitment problems also resulted in a wider age range, including participants older than the intended age range of the CDI questionnaire used to assess depressive symptoms (Kovacs, 2001). Adjusting the wording of four items (see method section) appeared to result in an age appropriate measure with good reliability, yet a measure developed for the entire age range would be preferable.

A further limitation is that our shortened and written adaptation of the life story interview to assess narrative identity (McAdams, 2008) had limited reliability and considerable missing data. Despite our intent to reduce participant burden, some participants skipped the items and the data was less detailed and rich impeding reliable coding. This makes any conclusions regarding narrative identity less reliable. The current study only showed a significant negative correlation between depressive symptoms and redemptive sequences at pre-test. Yet, previous research suggests that redemptive sequences and agency are related to lower depressive symptoms over time using assessments with better reliability (McAdams et al., 2001; Adler et al., 2015).

## Conclusion

In conclusion, the pursuit of alternative methods and action mechanisms for the prevention of depression remains valuable despite the lack of effectiveness of the commercial game in this study. Future research aimed at developing and/or testing mental health games are advised to use research designs better suited to initial exploration of action mechanisms and associated game mechanics. Ultimately, the promise of mental health video games — effective prevention tools that youth seek out and stay engaged in —can only be realized when engagement and strength of action mechanisms are both optimized.

## Data Availability Statement

The datasets presented in this study can be found in online repositories. The data is available at the DANS EASY online data repository as ‘Poppelaars et al. (2020) at https://doi.org/10.17026/dans-zhq-2qmc’.

## Ethics Statement

The study involving human participants was reviewed and approved by Ethical Committee of the Faculty of Social Sciences at Radboud University. Written informed consent to participate in this study was provided by the participant and when applicable by the participant and the participants’ legal guardian/next of kin.

## Author Contributions

MP, AL-A, and IG contributed to the conception and design of the study. MP coordinated and contributed to data collection, performed the statistical analyses in consultation with RO, and wrote the first draft of the manuscript. All authors contributed to data interpretation, critical manuscript revision, and read and approved the submitted version.

## Conflict of Interest

The authors declare that the research was conducted in the absence of any commercial or financial relationships that could be construed as a potential conflict of interest.

## Footnotes

1 https://youtu.be/61DZC-60x20

2 This strategy excluded 904 youth that did not report elevated depressive symptoms (*n* = 755; CDI < 13; Patient Health Questionnaire 2 ≥ 2) or core depressive symptoms (*n* = 149; CDI ≥ 13; Patient Health Questionnaire 2 < 2).

3 https://youtu.be/s1oZnf3475c
