# Supplementary material for: Can a Commercial Video Game Prevent Depression? Null Results and Whole Sample Action Mechanisms in a Randomized Controlled Trial
Source: Front Psychol. 2021 Jan 12;11:575962. doi: 10.3389/fpsyg.2020.575962 (PMC7835124; doi:10.3389/fpsyg.2020.575962)
Supplement: Supplementary file 1 [file Table_1.pdf]

Supplementary Material

1 Tables

Table S1. Overview of Outcomes, Action Mechanisms, and Game Engagement Variables Assessed in the Randomized Controlled Trial Testing the Effectiveness of Journey.

	Screening	Pre-test	Post-test	6-month follow-up	12-month follow-up
Main outcome					
Depressive symptoms	●	●	●	●	●
Action mechanisms					
Rejection sensitivity		●	●	●	●
Narrative identity					
Redemptive meaning		●	●	●	●
Agency		●	●	●	●
Hope and optimism		●	●	●	●
Coping strategies					
Rumination		●	●	●	●
Distraction and problem solving		●	●	●	●
Game engagement[a]					
Intrinsic motivation			●		
Psychological need satisfaction					
Autonomy			●		
Competence			●		
Flow			●		
Excluded secondary outcome					
Core depressive symptoms	●	●	●	●	●
Excluded action mechanisms					
Coping competence		●	●	●	●
Self-esteem		●	●	●	●
Dependency		●	●	●	●
Excluded game engagement					
Logbook of game experience[a]			●		
Psychological need satisfaction					
Relatedness[b]			●		
Humanness other player[b]			●		

Note. [a] Assessed only for the Journey and Flower conditions. [b] Assessed only for the Journey condition.

Table S2. Descriptives (Means and Standard Deviations) and F-values for Depressive Symptoms, Action Mechanisms, and Game Engagement Variables for the Total Sample and per Condition.

	Total		Journey		Flower		Passive control		*df*	*F*
	M	*(SD)*	*M*	*(SD)*	*M*	*(SD)*	*M*	*(SD)*		
T0 DS	17.27	(3.75)	17.48	(3.78)	17.20	(3.99)	17.15	(3.51)	2, 241	0.18
T1 DS	15.95	(5.47)	15.77	(5.65)	15.85	(5.51)	16.24	(5.30)	2, 241	0.18
T2 DS	15.88	(6.13)	15.43	(6.22)	15.86	(6.25)	16.33	(5.97)	2, 233	0.42
T3 DS	14.65	(6.83)	14.22	(6.65)	14.34	(6.81)	15.38	(7.03)	2, 224	0.66
T4 DS	14.25	(6.96)	14.43	(7.24)	13.97	(6.29)	14.35	(7.39)	2, 223	0.09
T1 RS	9.58	(4.45)	9.75	(4.04)	9.14	(4.25)	9.85	(5.02)	2, 241	0.59
T2 RS	9.28	(4.50)	9.25	(4.23)	9.23	(4.57)	9.35	(4.74)	2, 232	0.01
T3 RS	8.81	(4.42)	9.31	(4.35)	8.44	(4.39)	8.70	(4.55)	2, 223	0.75
T4 RS	8.62	(4.73)	8.75	(4.88)	8.26	(4.37)	8.85	(4.95)	2, 219	0.33
T1 NR	1.21	(1.29)	1.19	(1.38)	1.32	(1.31)	1.12	(1.18)	2, 228	0.48
T2 NR	1.06	(1.13)	1.15	(1.08)	1.06	(1.10)	0.99	(1.20)	2, 198	0.36
T3 NR	1.06	(1.24)	1.05	(1.35)	1.05	(1.09)	1.08	(1.27)	2, 191	0.02
T4 NR	1.31	(1.43)	1.38	(1.47)	1.16	(1.25)	1.39	(1.57)	2, 189	0.53
T1 NA	1.58	(1.03)	1.51	(1.07)	1.65	(1.05)	1.59	(0.98)	2, 228	0.39
T2 NA	1.49	(1.02)	1.50	(1.01)	1.41	(1.04)	1.55	(1.00)	2, 198	0.33
T3 NA	1.43	(1.10)	1.32	(1.11)	1.50	(1.15)	1.48	(1.04)	2, 191	0.55
T4 NA	1.51	(1.04)	1.44	(1.01)	1.58	(0.97)	1.51	(1.15)	2, 190	0.29
T1 H&O	2.49	(0.44)	2.53	(0.43)	2.47	(0.44)	2.47	(0.44)	2, 241	0.53
T2 H&O	2.47	(0.50)	2.52	(0.53)	2.45	(0.44)	2.43	(0.54)	2, 232	0.76
T3 H&O	2.53	(0.50)	2.55	(0.48)	2.53	(0.50)	2.51	(0.53)	2, 224	0.10
T4 H&O	2.60	(0.51)	2.55	(0.50)	2.64	(0.49)	2.61	(0.54)	2, 220	0.56
T1 RUM	18.50	(7.49)	18.51	(7.58)	17.34	(7.16)	19.62	(7.64)	2, 241	1.90
T2 RUM	18.63	(7.93)	18.89	(8.59)	17.76	(7.80)	19.25	(7.40)	2, 232	0.76
T3 RUM	17.99	(8.04)	17.89	(8.60)	17.82	(7.81)	18.26	(7.79)	2, 223	0.07
T4 RUM	17.98	(7.91)	17.73	(8.36)	18.85	(7.68)	17.39	(7.73)	2, 219	0.68
T1 D&P	8.51	(3.99)	8.30	(4.00)	8.56	(4.11)	8.66	(3.91)	2, 241	0.17
T2 D&P	8.34	(3.86)	7.87	(3.84)	8.37	(4.07)	8.76	(3.65)	2, 232	1.05
T3 D&P	8.60	(4.12)	8.11	(4.03)	8.80	(4.26)	8.87	(4.07)	2, 223	0.78
T4 D&P	8.98	(4.12)	8.32	(3.88)	9.18	(3.86)	9.43	(4.56)	2, 219	1.50
IM	4.18	(1.62)	4.73	(1.53)	3.66	(1.54)			1, 151	**18.40*** **
AT	3.93	(1.59)	4.48	(1.49)	3.40	(1.52)			1, 151	**19.85*** **
CM	4.18	(1.56)	4.67	(1.37)	3.71	(1.60)			1, 151	**15.59*** **
FL	4.44	(2.20)	4.96	(2.20)	3.94	(2.10)			1, 151	**8.60** **

Note. Significant effects are printed in bold.
DS = Depressive Symptoms; RS = Rejection Sensitivity; NR = Narrative Redemptive Sequences; NA = Narrative Agency; H&O = Hope and Optimism; RUM = Rumination; D&P = Distraction and Problem Solving; IM = Intrinsic Motivation; AT = Autonomy; CM = Competence; FL = Flow.
** = p < .01. *** = p < .001.
